# CircSMAD4 alleviates high glucose-induced inflammation, extracellular matrix deposition and apoptosis in mouse glomerulus mesangial cells by relieving miR-377-3p-mediated *BMP7* inhibition

**DOI:** 10.1186/s13098-021-00753-1

**Published:** 2021-11-20

**Authors:** Rina Wu, Zheli Niu, Guangwei Ren, Lin Ruan, Lijun Sun

**Affiliations:** 1 Department of Endocrinology, Affiliated Hospital of Inner Mongolia University for Nationalities, Tongliao, China; 2 Department of Nephrology, The First Hospital of Hebei Medical University, 9 Donggang Road, Shijiazhuang City, 050030 Hebei Province China

**Keywords:** circSMAD4, miR-377-3p, *BMP7*, Diabetic nephropathy

## Abstract

**Background:**

Diabetic nephropathy (DN) is a common complication of diabetes mellitus. Accumulating studies suggest that the deregulation of circular RNA (circRNA) is involved in DN pathogenesis. This study aimed to investigate the role of circSMAD4 in DN models.

**Methods:**

Mice were treated with streptozotocin to establish DN models in vivo. Mouse glomerulus mesangial cells (SV40-MES13) were treated with high glucose to establish DN models in vitro. The expression of circSMAD4, miR-377-3p and bone morphogenetic protein 7 (*BMP7*) mRNA was measured by quantitative real-time PCR (qPCR). The releases of inflammatory factors were examined by ELISA. The protein levels of fibrosis-related markers, apoptosis-related markers and *BMP7* were checked by western blot. Cell apoptosis was monitored by flow cytometry assay. The predicted relationship between miR-377-3p and circSMAD4 or *BMP7* was validated by dual-luciferase reporter assay or pull-down assay.

**Results:**

CircSMAD4 was poorly expressed in DN mice and HG-treated SV40-MES13 cells. HG induced SV40-MES13 cell inflammation, extracellular matrix (ECM) deposition and apoptosis. CircSMAD4 overexpression alleviated, while circSMAD4 knockdown aggravated HG-induced SV40-MES13 cell injuries. MiR-377-3p was targeted by circSMAD4, and miR-377-3p enrichment partly reversed the effects of circSMAD4 overexpression. *BMP7* was a target of miR-377-3p, and circSMAD4 regulated *BMP7* expression by targeting miR-377-3p. MiR-377-3p overexpression aggravated HG-induced injuries by suppressing *BMP7*.

**Conclusion:**

CircSMAD4 alleviates HG-induced SV40-MES13 cell inflammation, ECM deposition and apoptosis by relieving miR-377-3p-mediated inhibition on *BMP7* in DN progression.

## Introduction

Diabetic nephropathy (DN), a serious complication of diabetes, is the most common cause of chronic kidney disease (CKD) and end-stage renal disease (ESRD) in the world [1]. In the past 20 years, the morbidity and mortality of DN have risen rapidly in the global population [2]. Clinically, DN manifests as increased urinary albumin excretion (> 300 mg/day), decreased glomerular filtration rate (GFR), diabetic glomerulopathy, and increased arterial blood pressure [3, 4]. Pathologically, there are many changes in the kidney, including extracellular matrix (ECM) deposition (mainly in the mesangium), thickening of the glomerular basement membrane, interstitial fibrosis, and glomerular sclerosis [5]. DN has significantly negative impacts on society in the fields of public health and socioeconomics. Therefore, more research needs to be carried out to clarify the pathogenesis of DN to prevent the disease and improve treatment.

Circular RNA (circRNA) has been a hot research topic in the field of medicine in recent years. As a member of non-coding RNAs, circRNA is famous for its circular closed structure, which makes them more stable compared to linear molecules [6]. CircRNA is widely expressed in tissues, cells and multiple body fluids, such as blood, urine and saliva [7]. CircRNAs have been recognized as promising biomarkers for disease diagnosis and management due to wide expression and easy-to-detect [6, 8]. Mounting studies identify that numerous circRNAs are implicated in the progression of DN. For example, Hu et al. established DN cell model by treating mouse mesangial cells (SV40-MES13) with high glucose (HG) and then found that circRNA_15698 promoted ECM accumulation in HG-induced SV40-MES13 cells [9]. Peng et al. discovered that circRNA_010383 suppressed HG-induced ECM accumulation and blocked proteinuria and renal fibrosis in DN mice [10]. A previous circRNA expression profile identified several differentially expressed circRNAs in kidney tissues from DN mice, and we noticed that mmu_circ_0000894 was poorly expressed in DN mice [11]. The data from circbase database display that mmu_circ_0000894 is derived from mouse SMAD4 gene by “backsplicing”, also named circSMAD4. However, the role of circSMAD4 in DN progression is unclear and needs to be further investigated.

As for molecular mechanism, numerous circRNAs were shown to serve as a competing endogenous RNA (ceRNA) to regulate gene expression by competing for microRNA (miRNA) binding site [12]. For instance, circRNA_15698 acted as miR-185 sponge to elevate *TGF-β1*, thus enhancing ECM accumulation in HG-treated SV40-MES13 cells [9]. The exploitation of bioinformatics databases makes it easy to predict the potential targets of circRNA or miRNA. Therefore, we hypothesize that circSMAD4 governs the miRNA/mRNA axis in DN development and further verify this.

In this study, we established DN mouse model and DN cell model and examined the expression of circSMAD4 in these models. Besides, we investigated the function of circSMAD4 on inflammatory responses, ECM accumulation and apoptosis in SV40-MES13 cells. Moreover, the interactions among circSMAD4, miR-377-3p and bone morphogenetic protein 7 (*BMP7*) were determined to provide a functional mechanism of circSMAD4.

## Materials and methods

### Animal models

C57BL/6 mice (female, 6-week-old) were purchased from Vital River Laboratory Animal (Beijing, China) and housed in pathogen-free conditions. These mice were divided into two groups (n = 6/group), control and diabetic mice (DM). Mice in the DM group were administered with high-fat and high-glucose diets. Then, mice were intraperitoneally injected with streptozotocin (45 mg/kg; Sigma-Aldrich, St. Louis, MO, USA) to increase the burden on the kidney for consecutive 7 days. Streptozotocin was dissolved in 0.1 M citrate acid solution. Mice were considered to have DN until fasting blood-glucose reached 16.5 mmol/L, urinary albumin level over 30 mg/24 h. Mice in the control group were administered with standard laboratory chow and injected with citrate acid solution (45 mg/kg) for consecutive 7 days. Mice were sacrificed after 0, 1, 2 and 4 months, and kidney tissues were excised for further analysis. Animal studies were performed in agreement with the guidelines of the Animal Care and Use Committee of Affiliated Hospital of Inner Mongolia University for Nationalities.

### Cells and cell treatment

Mouse glomerulus mesangial cells (SV40-MES13; Procell, Wuhan, China) were cultured in 71.25% DMEM + 23.75% Ham’s F-12 medium + 5% FBS. Cells were administered with normal glucose (NG; Sigma-Aldrich; 5.5 mmol/mL), high glucose (HG; 25 mmol/mL) or mannitol (20 mmol/L; Sigma-Aldrich) and then cultured for 24 h. Cells were cultured at 37 °C conditions containing 5% CO_2_.

### Cell transfection

CircSMAD4 overexpression vector (circSMAD4), blank pCD-ciR expression vector (vector), short hairpin RNA targeting circSMAD4 (sh-circSMAD4), and its negative control (sh-NC) were all obtained from Geneseed (Guangzhou, China). MiR-377-3p mimic (miR-377-3p), miR-377-3p inhibitor (anti-miR-377-3p) and their corresponding negative controls (miR-NC and anti-NC) were bought from Ribobio (Guangzhou, China). *BMP7* overexpression vector (*BMP7*) and blank pcDNA expression vector (pcDNA) were provided by Genepharma (Shanghai, China). SV40-MES13 cells were transfected with oligos or vectors using Lipofectamine 3000 (Invitrogen, Carlsbad, CA, USA).

### ELISA

24 h mouse urine was collected to determine albumin and 8-OH-dG using the Mouse Albumin Detection ELISA Kit (Chondrex, Redmond, WA, USA) and QuickDetect 8-OH-dG (Mouse) ELISA Kit (BioVision, Milpitas, CA, USA) according to the protocols. The releases of inflammatory factors, including IFN-γ, MCP-1, IL-6 and TNF-α, were determined using the Mouse IFN-γ ELISA Kit (Sigma-Aldrich), MCP-1 (Mouse) ELISA Kit (BioVision), IL-6 (Mouse) ELISA Kit (BioVision) and TNF-α (Mouse) ELISA Kit (BioVision) in line with the instructions.

### Western blot

Total protein was extracted using RIPA lysis reagent (Cwbio, Beijing, China) and quantified by BCA kit (Cwbio). Equal amount of protein was loaded in 10% SDS-PAGE and then transferred on PVDF membranes, followed by blocking using 5% skim milk. The primary antibodies (Abcam, Cambridge, MA, USA), including anti-fibronectin (ab268021; 1/1000 dilution), anti-collagen IV (ab227616; 1/1000 dilution), anti-Bcl-2 (ab182858; 1/2000 dilution), anti-Bax (ab32503; 1/5000 dilution), anti-BMP7 (ab129156; 1/5000 dilution) and anti-β-actin (ab8226; 1/2000 dilution), were used to incubate the membranes overnight at 4 °C Next, the membranes were incubated with the secondary antibody. The blots on the membranes were presented using the ECL kit (Cwbio).

### Flow cytometry assay

SV40-MES13 cells were collected at 48 h post-transfection to determine the number of apoptotic cells using the FITC Annexin V Apoptosis Detection Kit (BD Biosciences, San Jose, CA, USA). According to the protocol, cells were resuspended in Annexin V-FITC binding buffer and next stained with Annexin V-FITC and propidium iodide (PI). The apoptotic cells were sorted and distinguished using a flow cytometer (BD Biosciences).

### Quantitative real-time PCR (qPCR)

Trizol reagent (Cwbio) was applied for total RNA isolation. Subsequently, cDNA was assembled using the PrimeScript 1st strand cDNA Synthesis Kit (Takara, Dalian, China) or microScript microRNA cDNA Synthesis Kit (Norgen Biotek, Thorold, Canada), followed by qPCR amplification using the SYBR reagent (Sigma-Aldrich). Using β-actin or U6 as the internal reference, the fold-change of relative expression was calculated using the 2^−ΔΔCt^ method. The primer sequences were shown as below:

circSMAD4 (mmu), F: 5′- CACTATGAGCGGGTTGTCTCA-3′ and R: 5′-AGCAGGATGACCATTACTCTGC-3′; miR-377-3p (mmu), F: 5′-CGCGATCACACAAAGGCAAC-3′ and R: 5′-AGTGCAGGGTCCGAGGTATT-3′; *BMP7* (mmu), F: 5′-CCTATGGCCATGTCGCATCT-3′ and R: 5′-GCAGCCCAAGCTACTGAAGA-3′; U6 (mmu), F: 5′-CTCGCTTCGGCAGCACATATACT-3′ and R: 5′-ACGCTTCACGAATTTGCGTGTC-3′; β-actin (mmu), F: 5′-CACTGTCGAGTCGCGTCC-3′and R: 5′-CGCAGCGATATCGTCATCCA-3′.

### Dual-luciferase reporter assay

MiR-377-3p was predicted as a target of circSMAD4 by starbase (http://starbase.sysu.edu.cn/). *BMP7* was predicted as a target of miR-377-3p by DIANA tools (http://diana.imis.athena-innovation.gr/DianaTools/index.php?r=site/index).

According to the wild-type (WT) sequence of circSMAD4 or *BMP7*, the mutant-type (MUT) sequence fragment of circSMAD4 or *BMP7* containing mutated miR-377-3p binding site was designed and synthesized. Then, the WT and MUT reporter vectors of circSMAD4 and *BMP7* were constructed, named WT-circSMAD4, MUT-circSMAD4, WT-*BMP7* 3′UTR and MUT-*BMP7* 3′UTR. Then, these reporter vectors were separately transfected into SV40-MES13 cells transfected with miR-377-3p or miR-NC. At 48 h post-transfection, luciferase activity in cells was tested using the dual-luciferase assay kit (Promega, Madison, WI, USA).

### RNA pull-down assay

SV40-MES13 cells were transfected with biotinylated miR-377-3p (Bio-miR-377-3p; 50 nM; Ribobio) or biotinylated miR-NC (Bio-miR-NC) and maintained for 24 h. The cells were then harvested and lysed in lysis buffer (Invitrogen). Cell lysates were incubated with streptavidin magnetic beads (Invitrogen) for 4 h. The beads were washed, and RNA compounds on beads were eluted and isolated for qPCR analysis.

### Statistical analysis

Data were collected from three independent experiments for each assay and operated using GraphPad Prism 7 (GraphPad, La Jolla, CA, USA). The differences in different groups were determined using Student’s *t*-test or using analysis of variance followed by the Tukey post-test. The data were displayed as the mean ± standard deviation (SD). *P* < 0.05 was considered to be statistically significant.

## Results

### CircSMAD4 was downregulated in DN mouse models

**Fig. 1:**
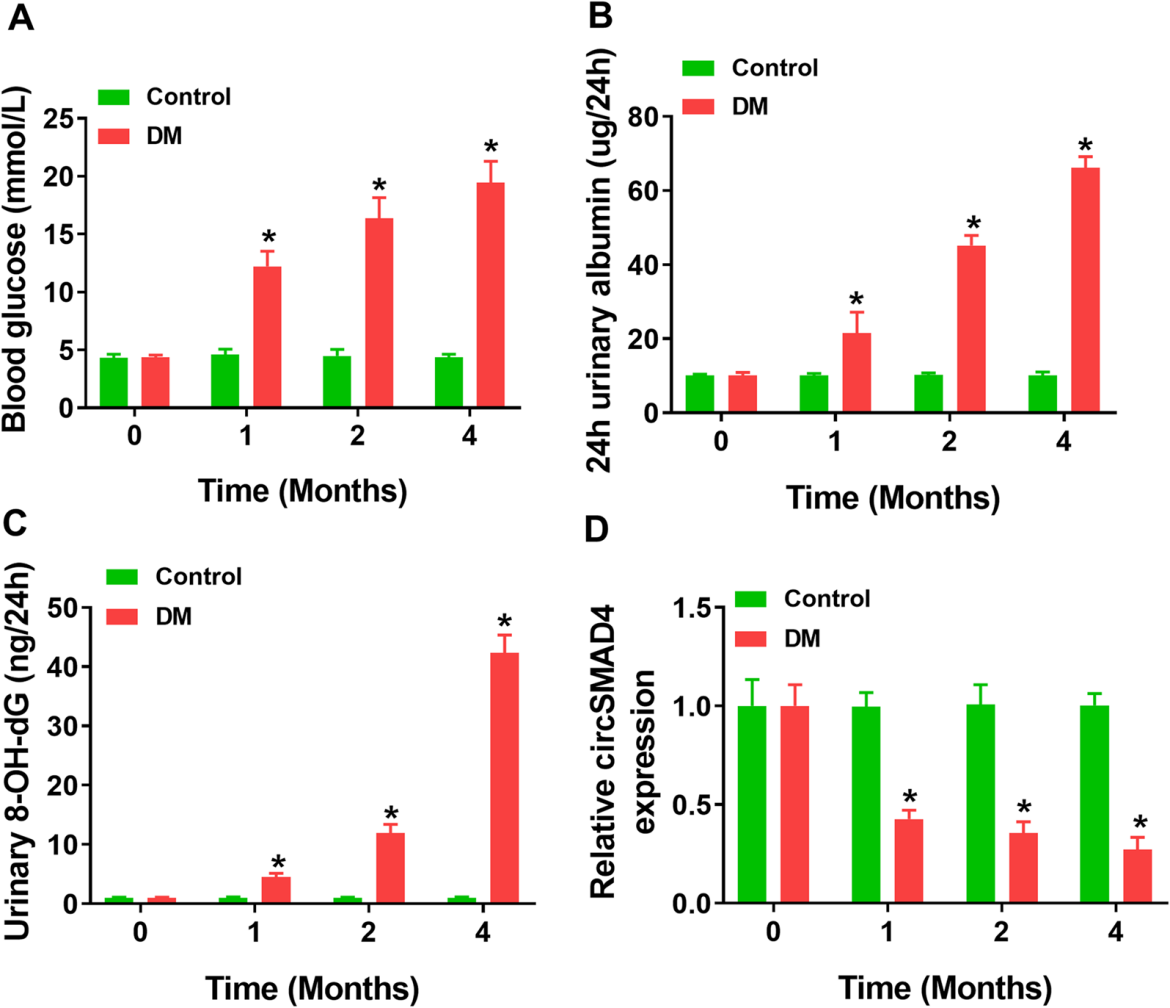
CircSMAD4 expression was decreased in DN models. **A** Blood glucose was measured in mice. **B** and **C** Urinary albumin and urinary 8-OH-dG were measured by ELISA. **D** The expression of circSMAD4 in kidney tissues was detected by qPCR. *P < 0.05

The data showed that the blood glucose of mice from DN group was significantly increased compared with that in control group (Fig. 1A). Besides, 24 h urinary albumin was strikingly enhanced in DN mice compared to control (Fig. 1B). Moreover, urinary 8-OH-dG was also markedly reduced in DN mice compared to control (Fig. 1C). Meanwhile, the expression of circSMAD4 was significantly declined in DN mice compared to control, and its expression was negatively correlated with the severity of DN (Fig. 1D). The data suggested that circSMAD4 was poorly expressed in DN mouse models.

### CircSMAD4 was downregulated in HG-treated SV40-MES13 cells, and HG promoted inflammation, cell apoptosis and ECM deposition

**Fig. 2:**
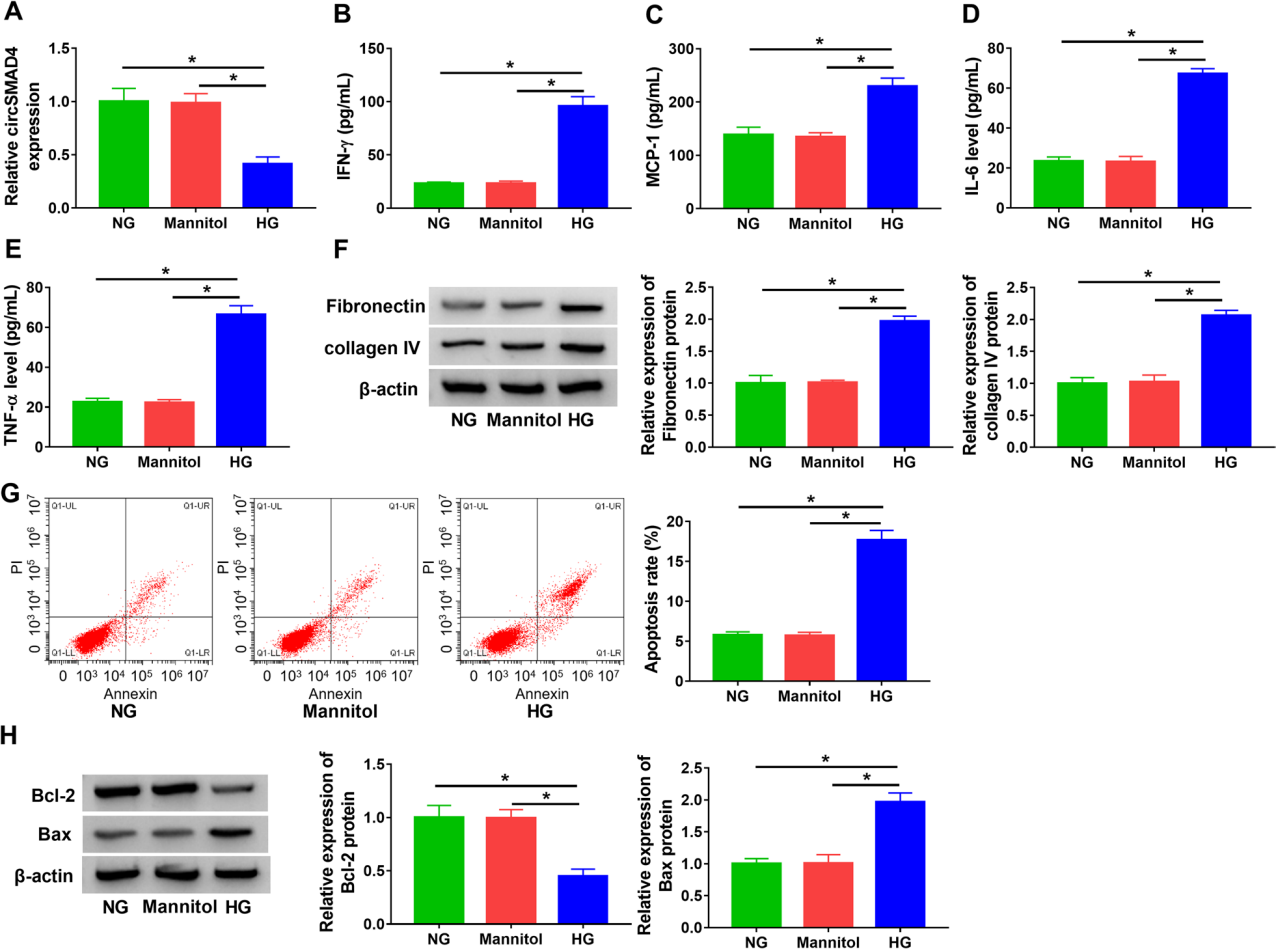
HG induced SV40-MES13 cell inflammation, ECM deposition and cell apoptosis. SV40-MES13 cells were treated with HG, NG or mannitol. **A** The expression of circSMAD4 was detected by qPCR. **B**–**E** The releases of IFN-γ, MCP-1, IL-6 and TNF-α in medium were determined by ELISA. **F** The protein levels of fibronectin and collagen IV were measured by western blot. **G** Cell apoptosis was assessed by flow cytometry assay. **H** The protein levels of Bcl-2 and Bax were measured by western blot. *P < 0.05

The expression of circSMAD4 was shown to be notably decreased in HG-treated SV40-MES13 cells compared with that in mannitol- or NG-treated cells (Fig. 2A). In function, HG largely promoted the secretion of pro-inflammatory factors, including IFN-γ, MCP-1, IL-6 and TNF-α (Fig. 2B–E). Besides, fibrosis-related proteins, including fibronectin and collagen IV, were shown to be upregulated in SV40-MES13 cells with HG treatment compared to NG or mannitol (Fig. 2F). In addition, flow cytometry assay presented that the number of apoptotic cells was strikingly increased in the HG-treated group compared to other groups (Fig. 2G). The expression of Bcl-2 was declined, while the expression of Bax was reinforced in HG-treated SV40-MES13 cells (Fig. 2H). These results manifested that HG triggered SV40-MES13 cell inflammation, ECM deposition and cell apoptosis.

### HG-induced SV40-MES13 cell inflammation, ECM deposition and cell apoptosis were alleviated by circSMAD4 overexpression

**Fig. 3:**
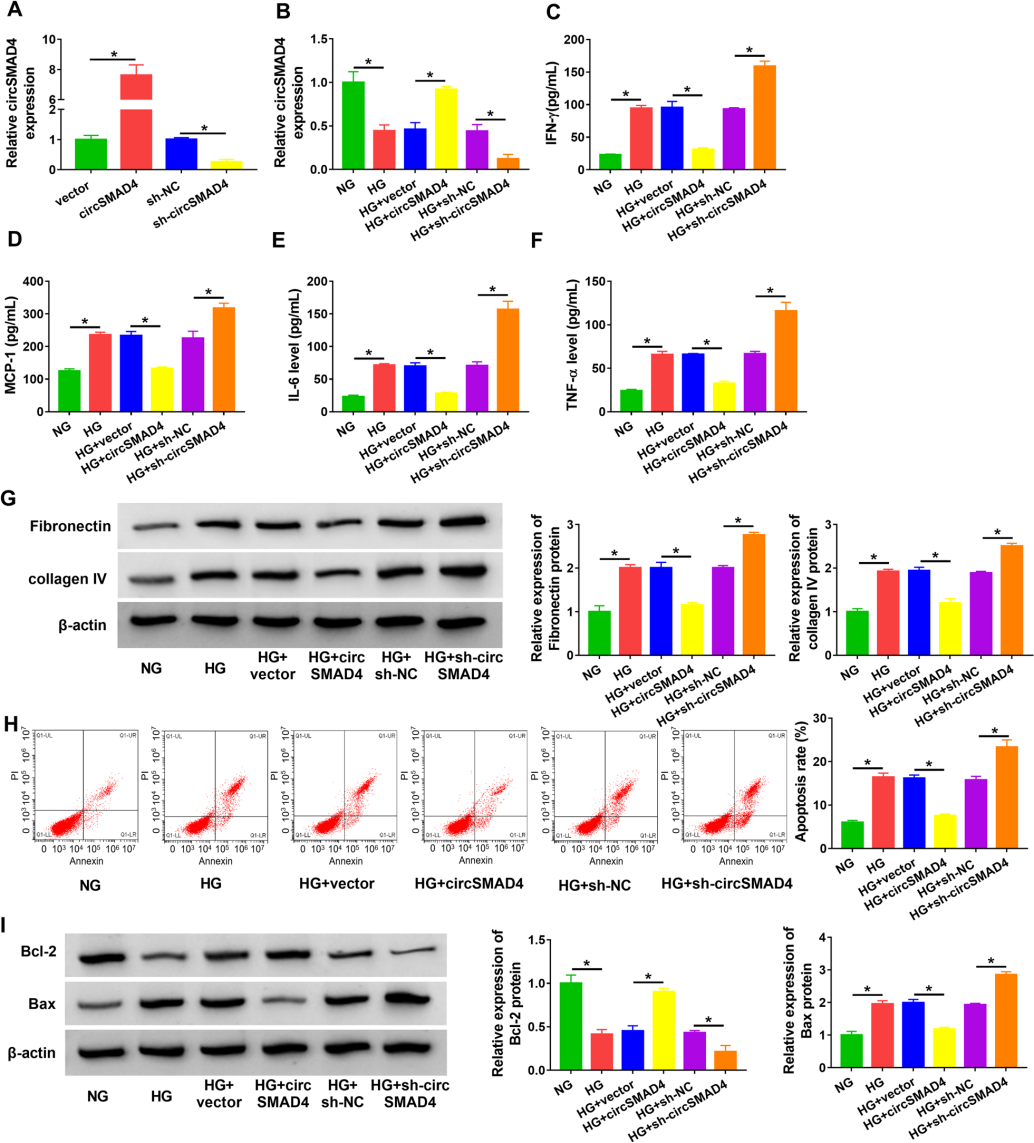
CircSMAD4 overexpression alleviated HG-induced SV40-MES13 cell inflammation, ECM deposition and cell apoptosis. **A** The efficiency of circSMAD4 overexpression or knockdown was checked by qPCR. HG-treated SV40-MES13 cells were transfected with circSMAD4 or sh-circSMAD4. Then, **B** the expression of circSMAD4 was detected by qPCR. **C**–**F** The releases of IFN-γ, MCP-1, IL-6 and TNF-α in medium were determined by ELISA. **G** The protein levels of fibronectin and collagen IV were measured by western blot. **H** Cell apoptosis was assessed by flow cytometry assay. **I** The protein levels of Bcl-2 and Bax were measured by western blot. *P < 0.05

Gain- and loss-function assays were performed to determine the role of circSMAD4 in HG-treated SV40-MES13 cells. The efficiency of circSMAD4 overexpression and knockdown was first checked, and the data showed that circSMAD4 expression was markedly enhanced in SV40-MES13 cells transfected with circSMAD4 but notably reduced in cells transfected with sh-circSMAD4 (Fig. 3A). The expression of circSMAD4 decreased in HG-treated SV40-MES13 cells was partly recovered by circSMAD4 transfection, while its expression was further suppressed by sh-circSMAD4 transfection (Fig. 3B). In function, the releases of IFN-γ, MCP-1, IL-6 and TNF-α induced by HG were effectively alleviated by circSMAD4 overexpression but further stimulated by circSMAD4 knockdown (Fig. 3C–F). The protein levels of fibronectin and collagen IV promoted by HG were largely suppressed by circSMAD4 overexpression but further strengthened by circSMAD4 knockdown (Fig. 3G). HG-induced SV40-MES13 cell apoptosis was largely alleviated by circSMAD4 overexpression but further promoted by circSMAD4 knockdown (Fig. 3H). Additionally, the protein level of Bcl-2 decreased in HG-treated SV40-MES13 cells was restored in HG-treated SV40-MES13 cells transfected with circSMAD4 but further declined in HG-treated SV40-MES13 cells transfected with sh-circSMAD4, while the protein level of Bax was opposite to Bcl-2 expression (Fig. 3I). These results suggested that circSMAD4 overexpression alleviated HG-induced SV40-MES13 cell inflammation, ECM deposition and cell apoptosis.

### MiR-377-3p was targeted by circSMAD4 and was upregulated in HG-treated SV40-MES13 cells

**Fig. 4:**
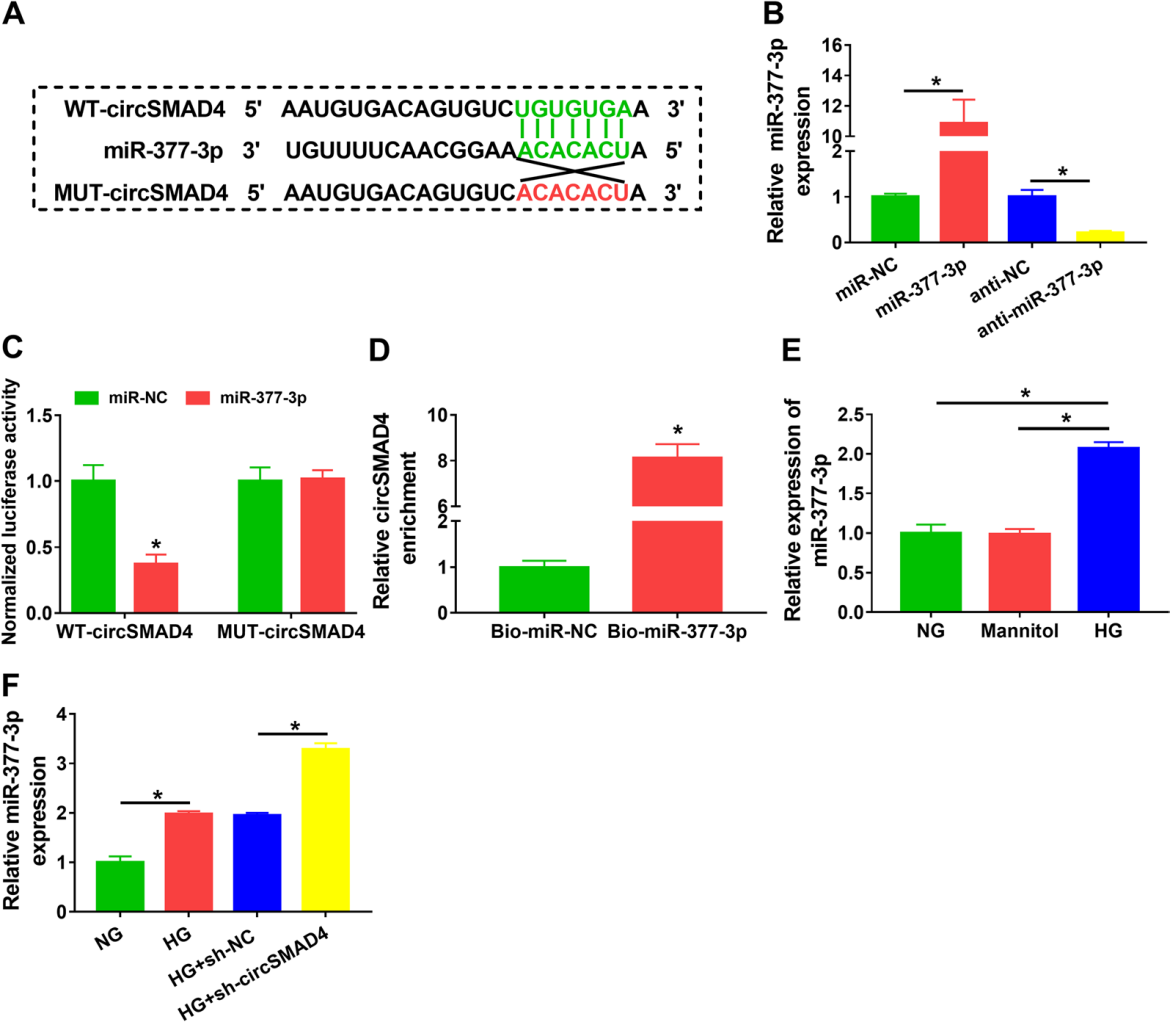
MiR-377-3p was a target of circSMAD4. **A** The binding relationship between circSMAD4 and miR-377-3p was predicted by starbase. **B** The efficiency of miR-377-3p overexpression or inhibition was checked by qPCR. **C** and **D** The binding relationship between circSMAD4 and miR-377-3p was verified by dual-luciferase reporter assay and pull-down assay. **E** The expression of miR-377-3p in SV40-MES13 cells was treated with HG, NG or mannitol was detected by qPCR. **F** MiR-377-3p expression in HG-treated SV40-MES13 cells with circSMAD4 absence was checked by qPCR. *P < 0.05

The target miRNAs of circSMAD4 were predicted by starbase, and miR-377-3p was shown to harbor the binding site with circSMAD4 (Fig. 4A). For miR-377-3p overexpression or inhibition, SV40-MES13 cells were transfected with miR-377-3p or anti-miR-377-3p, and the data presented that miR-377-3p expression was notably increased in cells transfected with miR-377-3p but notably decreased in cells transfected with anti-miR-377-3p (Fig. 4B). The data from dual-luciferase reporter assay showed that miR-377-3p overexpression significantly reduced luciferase activity in SV40-MES13 cells transfected with WT-circSMAD4 (Fig. 4C). Moreover, the data from pull-down assay presented that the expression of circSMAD4 could be notably enriched by Bio-miR-377-3p probe (Fig. 4D). The expression of miR-377-3p was remarkably enhanced in HG-treated SV40-MES13 cells compared to NG or mannitol (Fig. 4E). Moreover, miR-377-3p expression was enhanced in HG-treated SV40-MES13 cells, and its expression was further enhanced in HG-treated SV40-MES13 cells with circSMAD4 absence (Fig. 4F). These data highlighted that miR-377-3p was a target of circSMAD4.

### CircSMAD4 alleviated HG-induced SV40-MES13 cell inflammation, ECM deposition and cell apoptosis by suppressing miR-377-3p expression

**Fig. 5:**
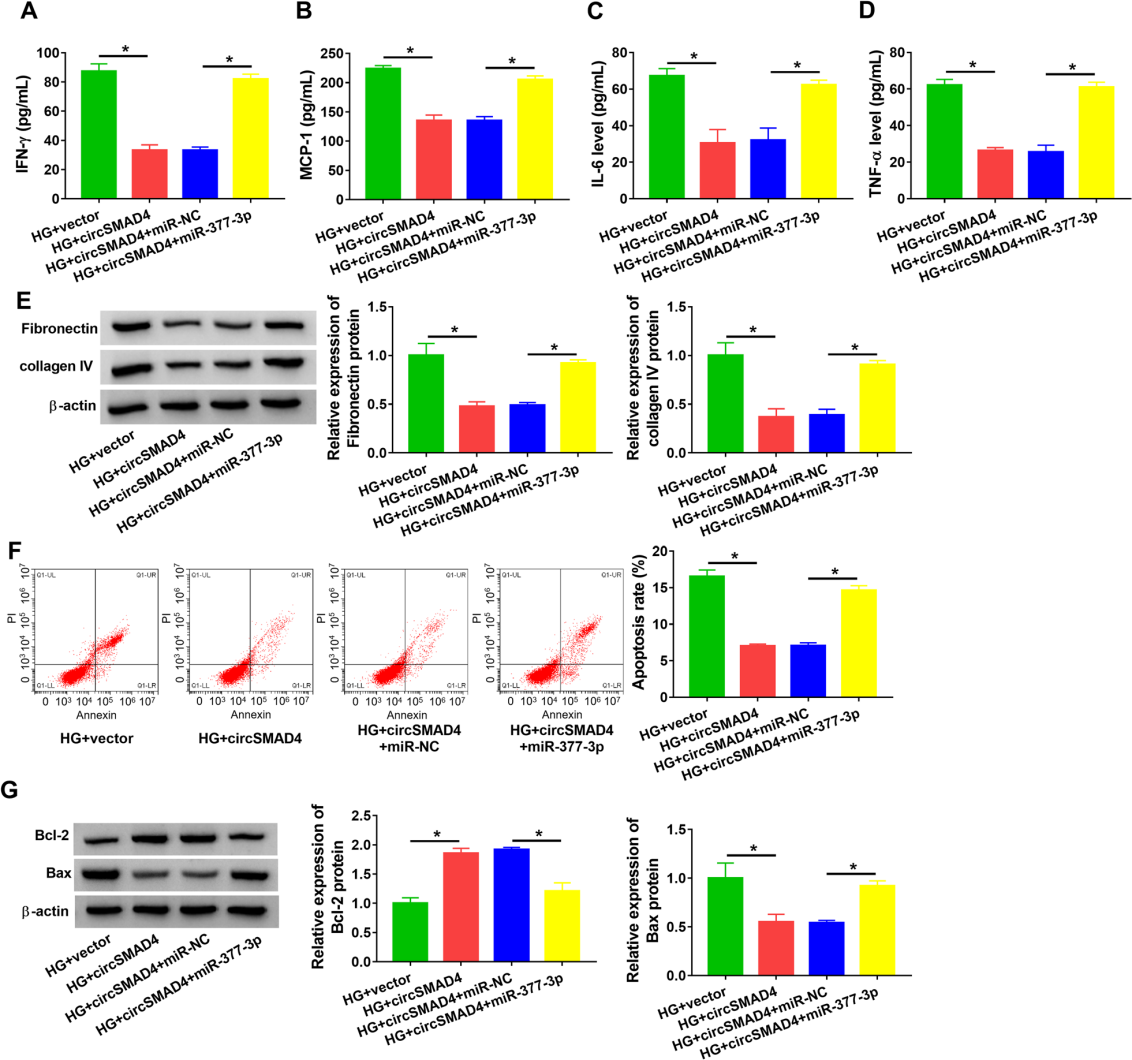
CircSMAD4 overexpression alleviated HG-induced SV40-MES13 cell inflammation, ECM deposition and cell apoptosis by impairing miR-377-3p expression. **A**–**G** SV40-MES13 cells treated with HG were transfected with circSMAD4 or circSMAD4 + miR-377-3p. **A–D** The releases of IFN-γ, MCP-1, IL-6 and TNF-α in medium were determined by ELISA. **E** The protein levels of fibronectin and collagen IV were measured by western blot. **F** Cell apoptosis was assessed by flow cytometry assay. **G** The protein levels of Bcl-2 and Bax were measured by western blot. *P < 0.05

HG-treated SV40-MES13 cells were transfected with circSMAD4 alone or circSMAD4 + miR-377-3p. In function, the releases of IFN-γ, MCP-1, IL-6 and TNF-α were inhibited in HG-treated SV40-MES13 cells transfected with circSMAD4 but largely recovered in HG-treated SV40-MES13 cells transfected with circSMAD4 + miR-377-3p (Fig. 5A–D). The protein levels of fibronectin and collagen IV were notably decreased in HG-treated SV40-MES13 cells transfected with circSMAD4 but partially restored in HG-treated SV40-MES13 cells transfected with circSMAD4 + miR-377-3p (Fig. 5E). The apoptotic rate of HG-treated SV40-MES13 cells was markedly blocked by circSMAD4 transfection but partly recovered by circSMAD4 + miR-377-3p cotransfection (Fig. 5F). The expression of Bcl-2 was enhanced in HG-treated SV40-MES13 cells transfected with circSMAD4 but repressed in HG-treated SV40-MES13 cells transfected with circSMAD4 + miR-377-3p, while the expression of Bax in these cells was opposite to Bcl-2 expression (Fig. 5G). These data revealed that circSMAD4 alleviated HG-induced SV40-MES13 cell inflammation, ECM deposition and cell apoptosis by suppressing miR-377-3p expression.

### CircSMAD4 regulated the expression of *BMP7* by targeting miR-377-3p

**Fig. 6:**
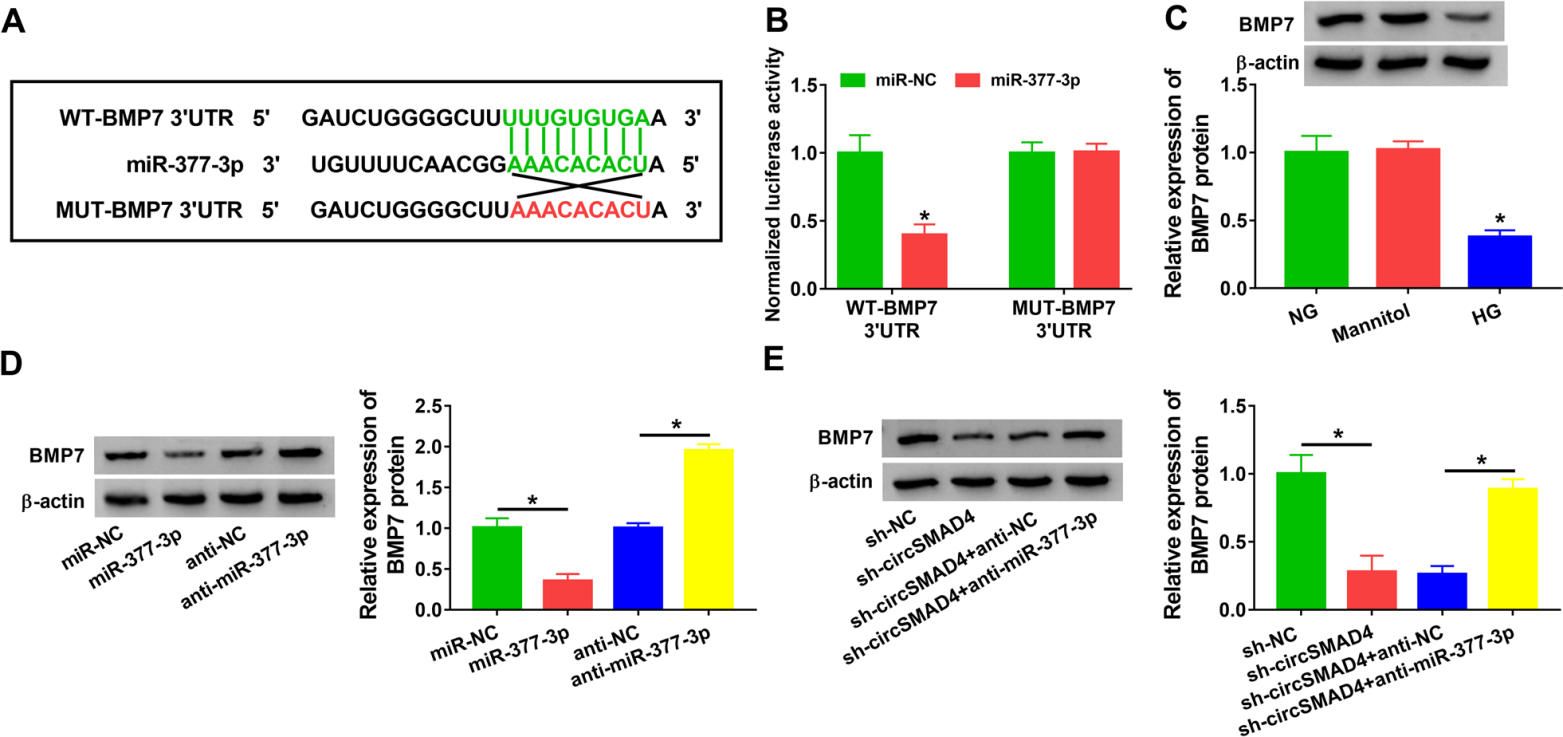
CircSMAD4 regulated *BMP7* expression by targeting miR-377-3p. **A** MiR-377-3p binding to *BMP7* 3′UTR was predicted by DIANA tools. **B** The interaction between miR-377-3p and *BMP7* was predicted by dual-luciferase reporter assay. **C** The expression of *BMP7* protein in SV40-MES13 cells treated with HG, NG or mannitol was detected by western blot. **D** The expression of *BMP7* protein in SV40-MES13 cells transfected with miR-377-3p or anti-miR-377-3p was measured by western blot. **E** The expression of *BMP7* protein in SV40-MES13 cells transfected with sh-circSMAD4 or sh-circSMAD4 + anti-miR-377-3p was detected by western blot. *P < 0.05

The target genes of miR-377-3p was predicted by DIANA tools, and miR-377-3p was shown to harbor binding site with *BMP7* 3′UTR (Fig. 6A). The data from dual-luciferase reporter assay displayed that miR-377-3p overexpression markedly weakened luciferase activity in SV40-MES13 cells transfected with WT-*BMP7* 3′UTR (Fig. 6B). The expression of *BMP7* protein was strikingly reduced in HG-treated SV40-MES13 cells (Fig. 6C). Moreover, the expression of *BMP7* protein was markedly decreased in SV40-MES13 cells transfected with miR-377-3p but markedly promoted in SV40-MES13 cells transfected with anti-miR-377-3p (Fig. 6D). Importantly, we found that the expression of *BMP7* protein was markedly declined in SV40-MES13 cells transfected with sh-circSMAD4, while its expression was largely recovered in SV40-MES13 cells transfected with sh-circSMAD4 + anti-miR-377-3p (Fig. 6E). The data suggested that circSMAD4 regulated the expression of *BMP7* by targeting miR-377-3p.

### MiR-377-3p overexpression promoted HG-induced SV40-MES13 cell inflammation, ECM deposition and cell apoptosis by depleting *BMP7*

**Fig. 7:**
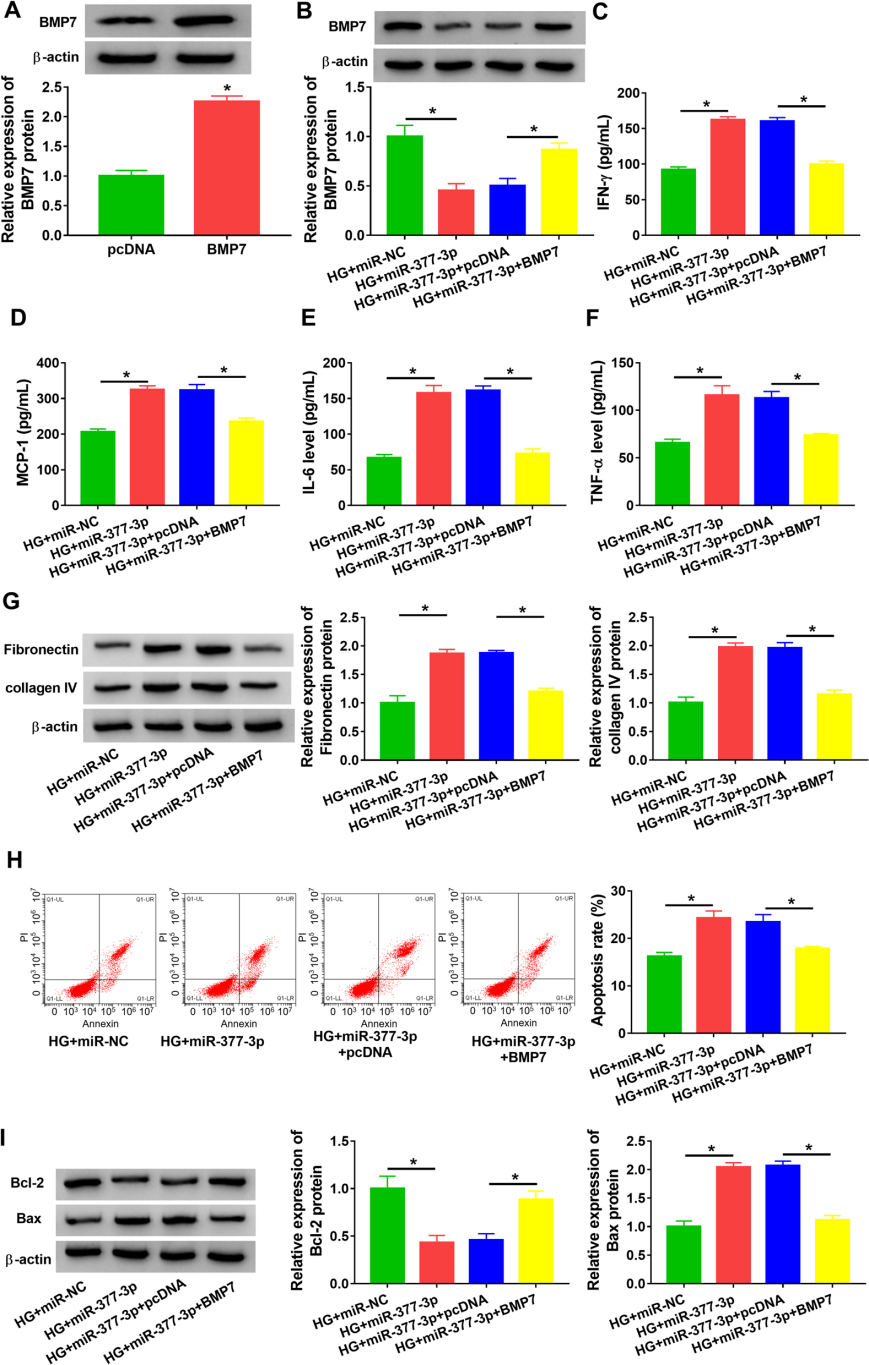
MiR-377-3p overexpression aggravated HG-induced SV40-MES13 cell inflammation, ECM deposition and cell apoptosis by suppressing *BMP7*. **A** The efficiency of *BMP7* overexpression was tested by western blot. **B**–**I** HG-treated SV40-MES13 cells were transfected with miR-377-3p or miR-377-3p + *BMP7*. **B** The expression of *BMP7* protein was detected by western blot. **C**–**F** The releases of IFN-γ, MCP-1, IL-6 and TNF-α in medium were determined by ELISA. **G** The protein levels of fibronectin and collagen IV were measured by western blot. **H** Cell apoptosis was assessed by flow cytometry assay. **I** The protein levels of Bcl-2 and Bax were measured by western blot. *P < 0.05

The expression of *BMP7* protein was markedly enhanced in SV40-MES13 cells transfected with *BMP7* compared to pcDNA (Fig. 7A). The expression of *BMP7* protein was notably decreased in HG-treated SV40-MES13 cells transfected with miR-377-3p but partially recovered in HG-treated SV40-MES13 cells transfected with miR-377-3p + *BMP7* (Fig. 7B). In function, the releases of IFN-γ, MCP-1, IL-6 and TNF-α were enhanced in HG-treated SV40-MES13 cells transfected with miR-377-3p but partly suppressed in HG-treated SV40-MES13 cells transfected with miR-377-3p + *BMP7* (Fig. 7C–F). The protein levels of fibronectin and collagen IV in HG-treated SV40-MES13 cells were strengthened by miR-377-3p transfection but repressed by miR-377-3p + *BMP7* transfection (Fig. 7G). HG-induced cell apoptosis was further enhanced by miR-377-3p overexpression but largely alleviated by the reintroduction of *BMP7* (Fig. 7H). The protein level of Bcl-2 in HG-treated SV40-MES13 cells was suppressed by miR-377-3p transfection but recovered by miR-377-3p + *BMP7* transfection, while the protein level of Bax was promoted by miR-377-3p transfection but largely repressed by miR-377-3p + *BMP7* transfection (Fig. 7I). These results suggested that circSMAD4-targeted miR-377-3p promoted HG-induced SV40-MES13 cell inflammation, ECM deposition and cell apoptosis by depleting *BMP7*.

## Discussion

Our study mainly discovered that circSMAD4 expression was strikingly decreased in DN mice and HG-treated SV40-MES13 cells. HG-induced SV40-MES13 cell inflammation, ECM accumulation and apoptosis were largely alleviated by circSMAD4 overexpression but further aggravated by circSMAD4 knockdown. Mechanism analysis revealed that circSMAD4 positively regulated *BMP7* expression by acting as miR-377-3p sponge. Accordingly, circSMAD4 governed the miR-377-3p/*BMP7* axis to prevent the progression of DN.

Mounting circRNAs are displayed to be aberrantly expressed in DN [13], and some of them are confirmed to be involved in DN pathogenesis, suggesting that circRNAs are novel class of regulators in DN. HG-treated mesangial cells are widely used as DN models in vitro [14–16]. Previous studies manifested that HG evoked inflammation, ECM accumulation and oxidative stress in mesangial cells [14]. Besides, HG was shown to induce mesangial cell injury by stimulating cell apoptosis, thus impairing cell functions [17]. We thus treated SV40-MES13 cells with HG and found that HG largely induced SV40-MES13 cell inflammation, ECM accumulation and apoptosis. CircSMAD4 was previously shown to be downregulated in DN mice by circRNA sequencing analysis [11]. Consistently, our study verified that circSMAD4 expression was remarkably reduced in streptozotocin-administered mice and HG-treated SV40-MES13 cells. Functional analysis uncovered that circSMAD4 overexpression largely alleviated HG-induced SV40-MES13 cell inflammation, ECM accumulation and apoptosis, while circSMAD4 knockdown aggravated these negative effects. Our study was the first to partly determine the role of circSMAD4 in DN and defined that circSMAD4 played a protective role in DN development.

To explore the regulatory mechanism of circSMAD4, we identified the target miRNAs of circSMAD4 and confirmed that miR-377-3p was a target of circSMAD4. MiR-377-3p was previously shown to be upregulated in DN mice and HG-treated mesangial cells, and it largely promoted the production of fibronectin and thus promoted mesangial cell fibrosis [18, 19]. MiR-377-3p was also proposed as a biomarker of DN [20]. Besides, miR-377-3p also gained considerable attention in inflammatory responses in human diseases [21, 22]. Consistent with these findings, miR-377-3p enrichment partly reversed the effects of circSMAD4 and thereby recovered HG-induced inflammation, ECM accumulation and apoptosis in SV40-MES13 cells, suggesting that miR-377-3p drove the development of DN. Our study clarified that miR-377-3p was involved in circSMAD4 network in DN.

Additionally, we showed that *BMP7* was a target gene of miR-377-3p. *BMP7* played an indispensable in DN. Wang et al. demonstrated that *BMP7* overexpression reversed diabetes-induced renal hypertrophy and recovered the function of glomerular filtration rate, urinary albumin excretion and glomerular histology [23]. Besides, forced *BMP7* expression inhibited glomerular fibrosis and interstitial collagen accumulation in diabetic mice [24]. In addition, *BMP7* overexpression attenuated oxidative stress and inflammatory responses in diabetic kidney disease [25]. In agreement with these findings, our results presented that the reintroduction of *BMP7* partly abolished the effects of miR-377-3p enrichment and effectively suppressed HG-induced inflammation, ECM accumulation and apoptosis in SV40-MES13 cells. Our study further determined that miR-377-3p inhibited the expression of *BMP7*, however, circSMAD4 acted as miR-377-3p sponge to relieve the inhibition of miR-377-3p on *BMP7*, indicating that circSMAD4 promoted the expression of *BMP7* by targeting miR-377-3p, thus preventing DN development.

Given that various cell types of glomerulus are all involved in DN pathogenesis, such as mesangial cells, podocytes and endothelial cells, the data regarding the functions of circSMAD4 in podocytes and endothelial cells are lacking in our present study. We only used mesangial cells as DN model in vitro, which was a main limitation. Future work should be prepared to address the role of circSMAD4 in podocytes and endothelial cells.

## Conclusion

Collectively, circSMAD4 was downregulated in DN models in vivo and in vitro. CircSMAD4 alleviated HG-induced inflammation, ECM deposition and apoptosis in mouse glomerulus mesangial cells by increasing *BMP7* expression via targeting miR-377-3p. We first proposed that circSMAD4 prevented the progression of DN partially by governing the miR-377-3p/*BMP7* pathway, aiming to provide a new strategy for DN treatment.

## Data Availability

Not applicable.
